# Green Infrastructure Design Based on Spatial Conservation Prioritization and Modeling of Biodiversity Features and Ecosystem Services

**DOI:** 10.1007/s00267-015-0613-y

**Published:** 2015-09-22

**Authors:** Tord Snäll, Joona Lehtomäki, Anni Arponen, Jane Elith, Atte Moilanen

**Affiliations:** Swedish Species Information Centre, Swedish University of Agricultural Sciences (SLU), PO 7007, 750 07 Uppsala, Sweden; Department of Biosciences, University of Helsinki, Viikinkaari 1, P.O. Box 65, 00014 Helsinki, Finland; School of Botany, The University of Melbourne, Parkville, Australia

**Keywords:** Green infrastructure, Corridor, Spatial conservation prioritization, Systematic conservation planning, Zonation software, Ecosystem services, Citizen science data

## Abstract

There is high-level political support for the use of green infrastructure (GI) across Europe, to maintain viable populations and to provide ecosystem services (ES). Even though GI is inherently a spatial concept, the modern tools for spatial planning have not been recognized, such as in the recent European Environment Agency (EEA) report. We outline a toolbox of methods useful for GI design that explicitly accounts for biodiversity and ES. Data on species occurrence, habitats, and environmental variables are increasingly available via open-access internet platforms. Such data can be synthesized by statistical species distribution modeling, producing maps of biodiversity features. These, together with maps of ES, can form the basis for GI design. We argue that spatial conservation prioritization (SCP) methods are effective tools for GI design, as the overall SCP goal is cost-effective allocation of conservation efforts. Corridors are currently promoted by the EEA as the means for implementing GI design, but they typically target the needs of only a subset of the regional species pool. SCP methods would help to ensure that GI provides a balanced solution for the requirements of many biodiversity features (e.g., species, habitat types) and ES simultaneously in a cost-effective manner. Such tools are necessary to make GI into an operational concept for combating biodiversity loss and promoting ES.

## The Need for Green Infrastructure

In many parts of the world, landscapes have become fragmented by habitat loss, resulting in increased distances between patches of semi-natural or natural habitats, decreased population sizes, and loss of species (Hanski 2011). The European continent has suffered more human-induced fragmentation than any other (Millennium Ecosystem Assessment 2001). For example, only 15 % of the forest species protected under the Habitats Directive have a favorable conservation status (European Commission 2012a). Under these circumstances, the EU has adopted a biodiversity strategy (European Commission 2012a) which includes targets to improve the conservation status of species and to strengthen green infrastructure within and across member states (European Commission 2012a).

There is a general agreement that green infrastructure will maintain and restore ecosystems, depending on the spatial structure of the management units and their management intensity (European Commission 2012a). However, very different approaches have been used to operationalize green infrastructure in land-use planning (European Environment Agency 2014; Kopperoinen et al. 2014). In the US, large, contiguous blocks of ecologically significant natural areas are linked with wide corridors to create an interconnecting network of natural lands across the landscape. For example, in Maryland, core forest areas constituting >100 ha were planned to be linked with corridors at least 350 m wide (Weber et al. 2006). In the fragmented continental Europe, green infrastructure has been related either to fine-scale urban applications aiming to identify corridors or biodiversity zones, or EU-scale compilations of coarse-grained spatial information (European Environment Agency 2014; Pauleit et al. 2011).

**Fig. 1:**
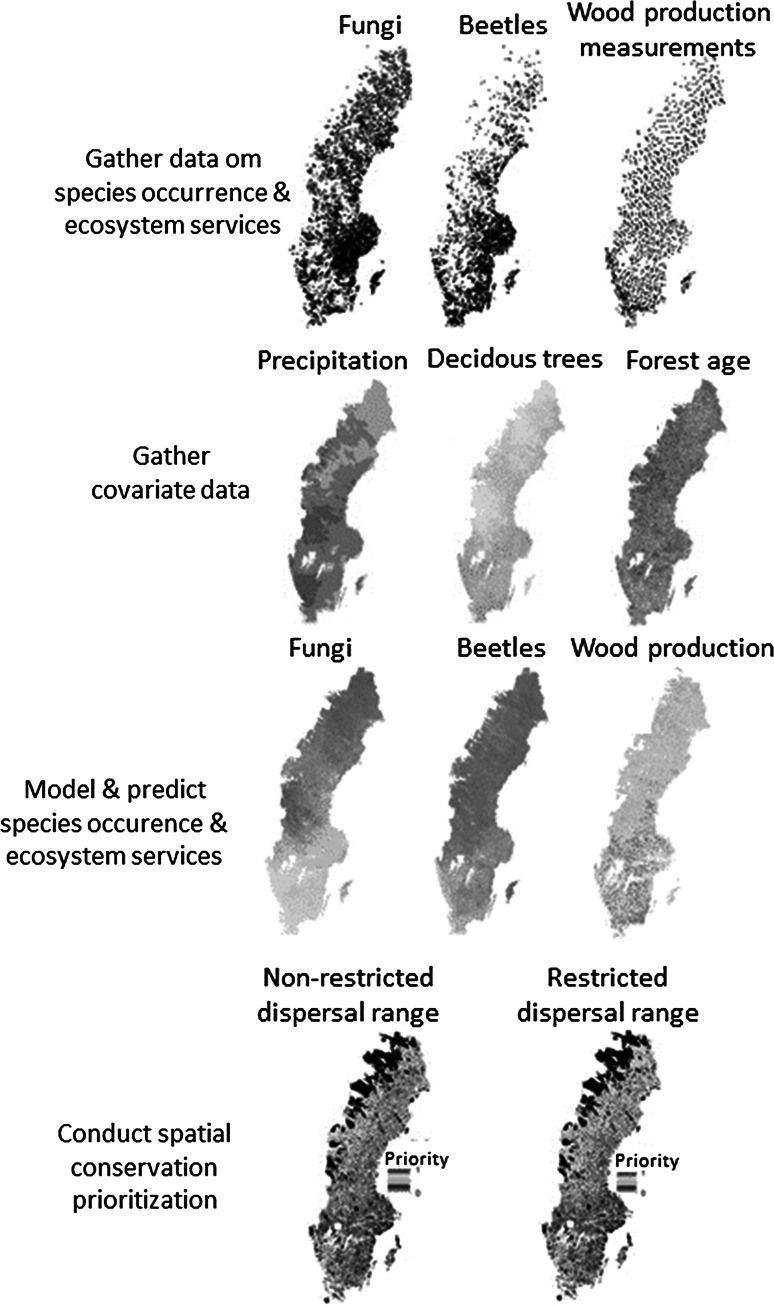
An approach for the design of green infrastructure. The first step is to gather data on occurrence of biodiversity features, including species, habitats, and ecosystem services (e.g., measured on national forest inventory plots). Second, gather predictor variables that are hypothesized to explain the distributions of the focal features. Third, model and predict the distribution of the features. Fourth, conduct spatial conservation prioritization using the model-predicted species and ecosystem service features in the same analysis. This optimization of the landscape from the perspective of species persistence and ecosystem service delivery may assume or ignore restricted species dispersal ranges

According to the EU (European Commission 2012a), green infrastructure has benefits beyond protecting biodiversity. It also promotes ecosystem services and the wellbeing of people, and has been attributed a role in the development of a green economy and sustainable land management. Green infrastructure design projects should thus also include ecosystem services representing different socio-economic interests (Cimon-Morin et al. 2013; European Environment Agency 2014; Kopperoinen et al. 2014; Maes et al. 2015) (Fig. 1).

Since green infrastructure is inherently a spatial concept, it should involve bringing together spatially explicit data and scientific spatial modeling and planning methods. We here provide our view on how current emerging data sources and scientific methods and concepts should be adopted for integrated planning for green infrastructure that accounts for biodiversity and ecosystem services. Notable, the recent report by the European Environment Agency (2014) about green infrastructure has not at all adopted the extensive scientific advances on these methods.

## Spatial Data and Models for Biodiversity and Ecosystem Services

For spatially explicit planning for green infrastructure, sufficiently accurate spatial data in relevant resolution are needed. These must include spatial data on ecosystem services and on the occurrence of multiple biodiversity features such as species and habitat types. With respect to species, it is well known that one taxonomic group is not necessarily a good surrogate for another, and that using solely abiotic environmental data to represent species distributions should be avoided (Rodrigues and Brooks 2007; Arponen et al. 2008). Therefore, data from many taxa are needed.

Although survey data at different resolutions are commonly available for many different taxa, they have limitations for detailed regional and local-level planning. For example, these types of data are often available on too coarse a scale (e.g., 10 × 10 km). Moreover, large-scale, high-resolution, and systematic observational data are typically lacking (e.g., for species that are listed in the Habitats Directive of the European Union).

Other types of species data are increasingly available. Species records from private or public natural history collections, check lists, inventories, and opportunistic sightings are now available on open-access websites, e.g., www.gbif.org or www.artportalen.se. The more recent records are often contributed by amateur observers (volunteers) therefore termed Citizen Science Data (CSD, Fig. 1, Schmeller et al. 2009; Silvertown 2009). These contributions are facilitated by the proliferation of handheld mobile devices (Teacher et al. 2013). Limitations of these data are well known, but they also have the potential to be useful (Yoccoz et al. 2001; Graham et al. 2004; Kery et al. 2010; Snäll et al. 2011).

Statistical species distribution models (SDMs) are routinely used to combine species and environmental data and provide continuous mapped predictions of habitat suitability (Elith and Franklin 2013 and Fig. 1). Diverse environmental variables can be used as predictor variables, and modeling methods are under continual development and include recent innovations that allow modelers to combine both survey data and opportunistically collected records (Dorazio 2014; Fithian et al. 2014).

In addition to species distributions, other kinds of data are relevant for the biodiversity component of green infrastructure. Distributions of habitat types can be modeled using a range of methods (Ferrier and Guisan 2006). Habitat mapping is also often based on interpretation of satellite imagery—for instance, see the Corine Land Cover mapping in Europe (http://www.epa.ie/soilandbiodiversity/soils/land/corine/). Mapped data on ecosystem services are also becoming increasingly available. The European Commission currently supports an online platform aiming at facilitating the sharing of ecosystem service maps and mapping methodologies (esp-mapping.net/), as part of the ongoing Mapping and Assessment of Ecosystems and their Services (MAES, Maes et al. 2014), which is one of the key actions of the EU biodiversity strategy (European Commission 2012a). Information about land-use patterns and publicly available interpretations of satellite imaging can also be used for developing regional maps of ecosystem services (Mulligan 2014; Kopperoinen et al. 2014; Snäll et al. 2014). Overlay analyses using Geographic Information Systems (GIS) have revealed that different parts of a landscape may be suitable for different bundles of ecosystem services (Raudsepp-Hearne et al. 2010). However, a key component of planning for green infrastructure or ecosystem services is to account for trade-offs and synergies among multiple ecosystem services in a spatially explicit context. It is not straightforward to do this solely by overlaying layers using a GIS (Bennett et al. 2009; Egoh et al. 2010; Reyers et al. 2012).

## Spatial Conservation Prioritization

During the last 20 years, there has been a strong methodological development of spatial conservation prioritization (SCP), which is a sub-discipline of conservation biology that uses computational methods and decision analysis in the allocation of protection or other conservation actions (Moilanen et al. 2009; Kukkala and Moilanen 2013). SCP can be utilized within the broader operational model of systematic conservation planning, which involves a set of steps for the engagement of stakeholders, data collection, target setting, analysis, and implementation of conservation (Margules and Sarkar 2007; Pressey and Botrill 2008). Since these steps are also key to green infrastructure design, SCP is a natural fit for the latter.

In SPC, the conservation priority of a spatial unit (raster cell, patch, etc.) is typically influenced by observed or model-predicted occurrences of biodiversity features, including species, habitat types, ecosystems, or ecosystem services (Kullberg and Moilanen 2014). Also relevant are costs, opportunity costs, alternative land-use needs, land ownership, and other types of (spatial) restrictions on the conservation solution. The priority of a spatial unit typically depends on the spatial configuration and connectivity of the landscape. The overall aim of these analyses often is to identify landscape structures that protect biodiversity locally and also facilitate landscape-level long-term-persistence of species.

SCP is well known for allowing users to identify valuable trade-offs and synergies between biodiversity and ecosystem services (Chan et al. 2006, 2011; Moilanen et al. 2011). More specifically, an initial step of SCP analysis is to give relative weights to the features accounted for, and these weights are used in the landscape optimization procedure. Weights have potentially large effects on the SCP solution (e.g., Moilanen et al. 2011) and it is therefore important to include experts and stakeholders regarding biodiversity and ecosystem services in green infrastructure design.

## Connectivity and Green Infrastructure: Corridors only a Partial Answer

Structurally continuous corridors are perhaps the most obvious means to connect green infrastructure throughout the landscape (Williams et al. 2005; Weber et al. 2006; Gilbert-Norton et al. 2010), as promoted by the European Environment Agency (2014). Corridors may be useful both at fine resolution to prescribe site-specific interventions, and at the coarse resolution, to maintain and facilitate movement, gene flow, range shifts, and other ecological and evolutionary processes that require large areas (Beier et al. 2011). In recent years, there has been strong emphasis on making corridors span environmental gradients to ensure that species can shift range distributions following climate change (e.g., Killeen and Solorzano 2008; Bernazzani et al. 2012). Also habitat restoration may be targeted within corridors (Rathore et al. 2012). Summarizing a large number of studies in a meta-analysis, Gilbert-Norton et al. (2010) recently found strong evidence that corridors increased movement between habitat patches by approximately 50 % compared to patches not connected with corridors, though the effect varied with distance between patches and taxa under consideration. They also found that natural corridors (those existing in landscapes prior to the study) enabled more movement than manipulated/restored corridors created for the purpose of the study.

Arguably, there are five issues that somewhat complicate the sole use of corridors for designing green infrastructure. First, many methods for corridor design rely on specification of resistance values to land cover types and subsequent analysis using least cost paths (LCP) or their multi-path extensions (e.g., Carroll et al. 2012; Rathore et al. 2012; Pinto et al. 2012; European Environment Agency 2014). It is a general problem associated with LCP analysis that the outcome can be highly sensitive to resistance values (Koen et al. 2012). Second, the resistance values are specific to a single species or a small group of species, which does not align well with an objective of designing green infrastructure for the benefit of all biodiversity and ecosystem services. Third, as a less significant constraint, corridor design methods typically require the end points of corridors to be specified a priori. Fourth, not all species or environments require structurally continuous connectivity such as wind-dispersed species for which other connectivity measures are more appropriate (e.g., Snäll et al. 2003). Fifth, sometimes corridors can act as attractive sinks that draw individuals away from breeding habitat into dispersal habitat, possibly even slowing down dispersal rates between habitat patches (Ovaskainen et al. 2008). This makes it clear that care needs to be practiced and experts need to be involved to make sure that corridor solutions are ecologically realistic, feasible, and advisable. As already stressed by Noss (1992), we should not allow corridors to substitute for the protection of large, intact core reserves or to divert attention from managing the landscape as a whole in an ecologically responsible manner.

## The Benefit of Spatial Conservation Prioritization for Green Infrastructure Design

Spatially explicit approaches are needed in the design of green infrastructure, because only they can support land managers’ decisions in real-world situations at the operational level (e.g., Millennium Assessment 2005). As detailed earlier, data and methods are available for predicting or mapping biodiversity features. Computational capacity for combining high-resolution SDM and SCP has been available for close to a decade now, following the development of tools such as Marxan or Zonation (Ball and Possingham 2000; Lehtomäki and Moilanen 2013 for recent references). Even though the European Commission (2012b) noted the potential for combining SDMs and SCP methods in the context of green infrastructure, applications are still lacking. The most recent approaches have been more conventional overlay analyses combining GIS data on, e.g., biodiversity occurrences and ecosystem services (Andersson et al. 2013; European Environment Agency 2014).

Maintaining ecosystem services is an additional goal of the EU (European Commission 2012a), and indeed, accounting for costs and human needs in SCP is usually essential for successful implementation (Pressey and Bottrill 2008; Cimon-Morin et al. 2013). As we have described, it is technically straightforward to integrate the increasingly available model-predicted ecosystem services (Maes et al. 2014) in the planning of green infrastructure using SCP methods (Chan et al. 2006; Moilanen et al. 2011). A key component of this SCP work will be to decide on the relative weights of, and trade-offs between biodiversity features and ecosystem services. For example, in Fennoscandia, there is a key trade-off between biodiversity and wood products, which are extracted on approximately 95 % of the productive forest land and is the main reason why many forest-dwelling species need a green infrastructure (Gärdenfors 2010). Biofuels are a particularly noteworthy type of resource use. Increased biofuel usage is promoted by the EU parliament with the goal to reduce the fossil carbon emissions (European Commission 2009). However, increased extraction of biofuel from the forest landscapes in the form of logging residues and stumps, which may provide habitats for red-listed species dependent on dead wood, may decrease the possibility to improve the conservation status of species in accordance with the EU biodiversity strategy (European Commission 2012a). Clearly, compromises will be needed when matching the needs of biodiversity, ecosystem services, and natural resource exploitation.

Although our view is that establishing large-scale corridors is not the key approach for green infrastructure design, also this can be achieved in the context of SCP. At simplest, one can develop the required corridors using any external method, and the locations of those corridors are then entered into SCP as a fixed part of the solution. Ideally though, one should develop corridor-building methods that simultaneously account for coverage of many biodiversity features, connectivity, costs, and other relevant factors (e.g., Rouget et al. 2006). To this effect, there have recently been steps towards the explicit integration of corridor building as part of the SCP process (Pouzols and Moilanen 2014).

Finally, knowledge of different experts and stakeholders regarding biodiversity and ecosystem services has a key role in the planning of conservation management, including design of the green infrastructure (Lehtomäki and Moilanen 2013). Combining qualitative information from experts with quantitative data in spatial conservation prioritization is not only required by the methods themselves, but also facilitates the uptake of scientific information by introducing concepts of SCP and green infrastructure to various stakeholders (Lehtomäki and Moilanen 2013; Kopperoinen et al. 2014).

## Conclusion

Green infrastructure design at different scales, from local through national to the EU-scale, is a major challenge, conceptually, in terms of data, and also in implementation. Use of state-of-the art methods will improve confidence in the quality of the outcome, thereby promoting public acceptability and increasing the likelihood of successful and well-balanced implementation (Possingham et al. 2006). Because green infrastructure by definition spans large geographic areas, some type of coordinated effort is needed in its design and implementation. Our view is that computational methods for statistical species distribution modeling, spatial conservation prioritization, and corridor approaches all include features that should be useful in the design of green infrastructure. Notably, these methods are absent from the recent report about green infrastructure by the European Environment Agency (2014). Development and maintenance of openly available national-scale biodiversity data is invaluable for enabling high-quality spatial planning for the benefit of the society at large, and thereby for the achievement of the policy goals for halting biodiversity loss.
